# Extracorporeal Membrane Oxygenation and Lung Transplantation: Initial Experience at a Single Brazilian Center

**DOI:** 10.6061/clinics/2020/e1698

**Published:** 2020-04-20

**Authors:** Flávio Pola-dos-Reis, Marcos Naoyuki Samano, Luis Gustavo Abdalla, Guilherme Vieira Soares de Carvalho, Lucas Matos Fernandes, Oswaldo Gomes-Júnior, Rafael Medeiros Carraro, Priscila Cilene Leon Bueno de Camargo, Ricardo Henrique Oliveira Braga Teixeira, José Eduardo Afonso-Júnior, Paulo Manoel Pêgo-Fernandes

**Affiliations:** I Programa de Transplante Pulmonar, Hospital Israelita Albert Einstein, Sao Paulo, SP, BR

**Keywords:** Lung Transplantation, Extracorporeal Membrane Oxygenation, Hypertension, Pulmonary, Pulmonary Fibrosis, Heart Failure, Diastolic

## Abstract

**OBJECTIVE:**

To report initial experience from the use of extracorporeal membrane oxygenation (ECMO) in patients who received lung transplantation.

**METHODS:**

Retrospective study of a single tertiary center in the Brazilian state of São Paulo, a national reference in lung transplantation, based on the prospective collection of data from electronic medical records. The period analyzed extended from January 2009 (beginning of the program) until December 2018.

**RESULTS:**

A total of 75 lung transplants were performed, with ECMO used in 8 (10.7%) cases. Of the patients, 4 (50%) were female. The mean age was 46.4±14.3 years. The causes of the end-stage lung disease that led to transplantation were pulmonary arterial hypertension in 3 (37.5%) patients, bronchiectasis in 2 (25%) patients, pulmonary fibrosis in 2 (25%) patients, and pulmonary emphysema in 1 (12.5%) patient. In our series, 7 (87.5%) cases were sequential bilateral transplantations. Prioritization was necessary in 4 (50%) patients, and in 1 patient, ECMO was used as a bridge to transplantation. The ECMO route was central in 4 (50%), peripheral venovenous in 2 (25%) and peripheral venoarterial in 2 (25%) patients. The mean length of the intensive care unit (ICU) stay was 14±7.5 days and of the hospital stay was 34.1±34.2 days. The mean ECMO duration was 9.3±6.6 days with a 50% decannulation rate. Three patients were discharged (37.5%).

**CONCLUSION:**

Lung transplantation requires complex treatment, and ECMO has allowed extending the indications for transplantation and provided adjuvant support in the clinical management of these patients.

## INTRODUCTION

Lung transplantation is a treatment alternative for end-stage lung diseases. According to the International Society of Heart and Lung Transplantation (ISHLT), the number of transplants and survival have both increased over the years 1. Additionally, patient complexity has also increased. During the same period, there has been a concomitant appearance of new technologies in thoracic surgery that have led to new patient support techniques, such as *ex vivo* lung perfusion 2, reconditioning of borderline lungs and donor lungs after cardiac death, and extracorporeal membrane oxygenation (ECMO) 3,4.

ECMO is a complex advanced life support technique that allows maintaining blood gas exchange (carbon dioxide removal and oxygenation) and long-term cardiocirculatory support, temporarily replacing the heart and lungs 5. ECMO in lung transplantation has been used for the treatment of primary graft dysfunction (PGD), as a bridge to transplantation and cardiocirculatory support during surgery, replacing extracorporeal circulation, to help manage hemodynamic instability in the postoperative period 3.

The objective of this study is to report the initial experience of the use of ECMO in a tertiary center that is a national reference for lung transplantation.

## MATERIALS AND METHODS

### Study design

This is a retrospective study of a single tertiary center, a national reference for lung transplantation, in the state of São Paulo, Brazil, based on the prospective collection of data from electronic medical records of said hospital. The period of analysis was from January 2009 (beginning of the program) to December 2018. This study was approved by the Research Ethics Committee of Hospital Israelita Albert Einstein, Brazil (approval number 22934919.5.0000.0071), and was performed in accordance with the ethical standards of the Brazilian National Research Ethics Commission and their subsequent amendments.

All patients undergoing lung transplantation who at some point needed ECMO were included. The investigated variables were the recipient characteristics, intraoperative data, and morbidity and mortality outcomes. There were no exclusion criteria. During this period, the surgical technique and immunosuppression protocol remained the same.

In all cases, ECMO was instituted by the hospital's lung transplant team, who was trained according to the recommendations of the Extracorporeal Life Support Organization (ELSO) 6. The team's perfusionists helped with adjusting the circuit parameters and controlling the activated coagulation time (ACT). Heparin was titrated to keep ACT between 180 and 240 seconds, and an additional blood product was used when necessary. Venovenous (VV) ECMO was instituted using the Seldinger technique, with the drainage cannula placed in the right common femoral vein and the return cannula in the right internal jugular vein. Peripheral venoarterial (VA) ECMO was instituted using the Seldinger technique, with the drainage cannula placed in the right common femoral vein and the return cannula in the left femoral artery. In all cases, we installed a distal perfusion cannula in the femoral artery. In central ECMO, the drainage cannula was placed in the right atrium and the return cannula in the ascending aorta. In these cases, the arterial flow was calculated based on the body surface area, with a target of 70% of the nominal flow, and was corrected, when necessary, based on the hemodynamic and metabolic demand during the procedure monitoring the arterial and venous pressure and the circuit pressure. The cell saver system was used during surgery. We used a centrifugal magnetic pump with a polymethylpentene oxygenator membrane (Rotaflow/Quadrox-ID, Getinge Cardiopulmonary AG, Hirrlinger, Germany). In all cases, the positions of the cannulae were checked by transesophageal echocardiography or chest X- ray/fluoroscopy.

### Statistical analysis

Quantitative data were analyzed using descriptive statistics and are expressed as means and standard deviations (±SD). Qualitative variables are expressed as frequencies and percentages. Statistical modeling and testing were performed with SPSS version 21.0.

## RESULTS

### Recipient profile

A total of 75 lung transplants were performed, of which ECMO was used in 8 (10.7%) cases. Of the patients, 4 (50%) were female. The mean age of the patients was 46.4±14.3 years, the mean time on the waiting list was 708.5±442.2 days, the mean systolic pulmonary artery pressure was 55.4±23.8 mmHg. The diagnoses of end-stage lung diseases that led to transplantation were pulmonary hypertension in 3 (37.5%) patients, bronchiectasis in 2 (25%) patients, pulmonary fibrosis in 2 (25%) patients, and pulmonary emphysema in 1 (12.5%) patient (Table 1).

### Lung transplantation characteristics

In our series, 7 (87.5%) cases were bilateral sequential transplants, of which 6 were left-right, with an ischemia time of the first side of 266.2±47.8 minutes and of the second side of 395±42.3 minutes. Prioritization (when we asked the state technical transplant agency to make priority for a transplant) was necessary in 4 (50%) patients, and in 1 patient, ECMO was used as a bridge to transplantation. The use of a cardiopulmonary bypass (CPB) was necessary in only 1 case (Table 1). The mean number of the transfused packed red blood cells was 1.7±1.2 bags. Tracheostomy was necessary in 4 (50%) patients.

### ECMO data

Regarding the time of ECMO initiation, it was used as a bridge to lung transplantation in only in 1 (12.5%) patient, during the surgery in 5 (62.5%) patients and postoperatively in 2 (25%) patients. The ECMO route was central in 4 (50%) patients, peripheral VV in 2 (25%) patients and peripheral VA in 2 (25%) patients. The ECMO route was changed in 3 (37.5%) patients; of the patients on central ECMO, 2 were switched to VV ECMO, after cardiocirculatory stability, and 1 was switched to VA ECMO and later to VV ECMO, for a total of 18 days on ECMO, after which the patient was decannulated. The mean ECMO duration was 9.3±6.6 days with a 50% decannulation rate, i.e., 4 patients. The mean length of the intensive care unit (ICU) stay was 14±7.5 days and of the hospital stay was 34.1±34.2 days. Regarding outcomes, 5 (62.5%) patients died during the same hospital stay, and 3 (37.5%) patients were discharged. Emergency ECMO was instituted in 2 (25%) cases. The reasons ECMO was required were pulmonary hypertension in 4 (50%) patients, hemodynamic instability in 2 (25%) patients, PGD in 1 (12.5%) patient and respiratory failure 1 (12.5%) patient. Renal replacement therapy was required in 6 (75%) cases. Complications secondary to ECMO were acute arterial occlusion requiring surgical intervention in 3 patients (37.5%), neurological events in 2 (25%) patients, deep venous thrombosis in 1 (12.5%) patient and major perioperative bleeding in 2 (25%) patients (Table 2).

## DISCUSSION

Lung transplantation is a treatment alternative for advanced lung diseases, such as obstructive, restrictive, and vascular lung disease and suppurations such as bronchiectasis. However, organ availability is scarce, and the recipient waiting list is growing. Because lung disease tends to be severe and progressive, increased waiting times for transplantation result in increased procedural morbidity and mortality 7.

In this context, ECMO as respiratory and/or cardiocirculatory support can be used as a tool to assist this population. The first use of ECMO in 1972 by Dr. Donald Hill as extracorporeal life support sparked the idea of using this technology 3. However, ECMO gained prominence in the treatment of acute respiratory distress syndrome (ARDS) in 2009 with the Conventional ventilation or ECMO for Severe Adult Respiratory failure (CESAR) Trial due to the H1N1 epidemic, and since then, there has been an increase in its use 8.

PGD, an early reperfusion acute lung injury in lung transplantation, mimics ARDS in cases of infection, and this phenomenon has an impact on morbidity and mortality in transplant recipients 9. PGD has an incidence between 15 and 57%, and 30-day mortality that can reach 24.5% in grade III PGD. The use of ECMO can be safely sustained until transplanted lungs have time to recover. In our series, ECMO was used in 1 (12.5%) patient for the treatment of PGD. The ECMO modality used is VV because the patient has respiratory failure but normal cardiac function 10.

ECMO can be used as a bridge to lung transplantation. Patients who are on the waiting list for transplantation can experience significant clinical worsening, leading to the inability of the lungs to perform their functions. In this case, ECMO is instituted to stabilize the clinical picture, and the patient is prioritized for transplantation 11. In our series, half of the patients were prioritized, of which ECMO was instituted as a bridge to lung transplantation in 1 (12.5%). One of the advantages of using ECMO as a bridge to lung transplantation is the possibility of maintaining it in place during and after surgery, guaranteeing ventilatory support and allowing protective mechanical ventilation 12.

Lung transplantation in patients with pulmonary hypertension poses challenges because there are technical difficulties and a higher rate of complications when compared to other patient groups. However, despite high mortality in the first year, lung transplantation offers a better long-term prognosis, worse only than in patients with cystic fibrosis 1. As a result of cardiac remodeling and right ventricular dysfunction, sometimes with compromised left ventricular function, bilateral or cardiopulmonary transplantation is the procedure of choice. Intraoperative cardiopulmonary care in these cases is mandatory, even in cases of bilateral sequential transplantation. One of the great advantages of ECMO over CPB is the possibility of being instituted before transplantation as a bridge and of being maintained after the procedure. Studies show that maintaining ECMO in the postoperative period in patients who underwent lung transplantation is associated with better survival, especially in the pulmonary arterial hypertension subgroup 13. Additionally, less anticoagulation and priming volume are necessary compared with those of conventional CPB. Studies have shown that ECMO is associated with a shorter length of ICU and hospital stays and lower rates of hemodialysis and reintubation compared with those of CPB 14, and currently, some transplant centers use ECMO as the first choice for circulatory care 15. In our series, 4 (50%) patients underwent transplantation due to pulmonary hypertension, of which the underlying diseases were idiopathic pulmonary hypertension in 2 patients, veno-occlusive pulmonary hypertension in 1 patient and pulmonary hypertension secondary to bronchiectasis due to cystic adenomatosis in 1 patient. In these cases, ECMO was initiated during the surgical procedure.

ELSO has developed guidelines that describe the ideal institutional requirements for the development of an ECMO program 6. Moll et al. described how to develop and implement an ECMO team in a hospital, motivated by unsatisfactory outcomes. After the implementation of the program, initial data showed that 53.3% of patients were decannulated 16, while in our service, the rate of decannulation was 50%. Regarding hospital discharge, only 40% of the patients in the study by Moll et al. were discharged from the hospital, whereas in our series, the rate was 37.5%; the mean ECMO duration was 4.8±3.9 days, and in our series, it was 14±7.5 days. A Korean study showed that the rate of postoperative decannulation failure after lung transplantation was 43.2%, causing longer ICU stays (16 days) than those in the decannulated group (6 days) (*p*<0.001); in the study, young donors with a high PO_2_/FiO_2_ ratio and short surgery time were factors that contributed to decannulation 17.

In our series, 6 (75%) patients required hemodialysis at some time during hospitalization. Banga et al. conducted a study using the UNOS database, correlating predictive factors for early hemodialysis after lung transplantation. Among these predictors, the use of preoperative ECMO (10.4%), mechanical ventilation (18.8%) and pulmonary hypertension (27.8%) were the most important 18. Another study using the UNOS database for patients who required ECMO after lung transplantation showed a greater risk for postoperative dialysis (hazard ratio [HR] 6.476, *p*<0.001) 19.

Studies have shown that ECMO has a lower risk of complications than does CPB. However, several complications are related to ECMO: neurological complications; bleeding at the puncture site; infection; bleeding such as digestive hemorrhage, hemothorax, and epistaxis; thrombocytopenia; and arterial occlusion. The most prevalent is bleeding, ranging from 5 to 79% in the literature. The cause is multifactorial but also secondary to iatrogenic anticoagulation. Arterial occlusion of the limb varies between 13-25% in the literature, while in our series, it was 37.5% 3,12,20. All patients underwent open embolectomy without other complications or sequelae.

## CONCLUSIONS

Lung transplantation requires complex treatment, and ECMO has allowed extending the indications for transplantation and provided adjuvant support in the clinical management of these patients. However, it is a technology that requires a team trained according to the guidelines of the governing organization because patients requiring ECMO are severely ill and manifest complications that, although treatable, must be strictly managed.

## AUTHOR CONTRIBUTIONS

Pola-dos-Reis F and Samano MN contributed to the conception of the study, data acquisition, analysis, and interpretation, and manuscript writing. Abdalla LG, Fernandes LM, Gomes-Júnior O, Carvalho GVS, Carraro RM, Camargo PCLB, Teixeira RHOB, Afonso-Júnior JE and Pêgo-Fernandes PM contributed equally to the conception and design of the study, and critical review of the manuscript for important intellectual content. All authors approved the final version of the manuscript and take public responsibility for the appropriate parts of the content.

## Figures and Tables

**Table 1:** Patient profile.

	Diagnosis	Cause for ECMO	Age at Transplantation (Years)	Sex (F/M)	Waitlist (days)	Modality of Transplant	Prioritization (Yes/No)	Bridge (Yes/No)	CPB (Yes/No)	Decannulation (Yes/No)	SPAP (mmHg)
Patient 1	Emphysema	PGD	60	F	634	Bilateral	No	No	No	No	35
Patient 2	Idiopathic Pulmonary Arterial Hypertension	Pulmonary Hypertension	56	M	119	Bilateral	No	No	No	No	51
Patient 3	Pulmonary Fibrosis	Hemodynamic Instability	56	M	202	Bilateral	Yes	No	No	No	50
Patient 4	Bronchiectasis	Pulmonary Hypertension	39	M	785	Bilateral	Yes	No	No	No	107
Patient 5	Idiopathic Pulmonary Arterial Hypertension	Pulmonary Hypertension	34	F	1555	Bilateral	No	No	No	Yes	107
Patient 6	Pulmonary Fibrosis	Hemodynamic Instability	65	F	727	Unilateral	Yes	No	Yes	Yes	40
Patient 7	Pulmonary Veno-Occlusive Disease	Pulmonary Hypertension	33	M	895	Bilateral	No	No	No	Yes	50
Patient 8	Cystic Fibrosis	Respiratory Failure	28	F	751	Bilateral	Yes	Yes	No	Yes	55

Abbreviations: PGD primary graft dysfunction; F female; M male; CPB cardiopulmonary bypass; SPAP systolic pulmonary arterial pressure.

**Table 2:** ECMO analysis.

	Route	Route Change	Time on ECMO	Dialysis (Yes/No)	Length at ICU	Length of Stay	30-day Mortality	1-year Mortality	Complication
			(Days)		(Days)	(Days)			
Patient 1	VV		8	Yes	24	24	Yes		Mesenteric ischemia
Patient 2	Central		2	No	2	2	Yes		Intraoperative bleeding and stroke
Patient 3	Central	2 PO VV	2	Yes	4	4	Yes		Sepsis
Patient 4	Central	16 PO VV	18	Yes	18	18	Yes		Stroke
Patient 5	VA Peripheral		5	Yes	14	115	No	Alive	Intraoperative bleeding/Occlusion arterial/Acute abdominal perforation
Patient 6	Central	VA/VV	18	Yes	18	18	Yes		Occlusion arterial
Patient 7	VA Peripheral		7	No	18	47	No	Alive	Occlusion arterial
Patient 8	VV		14	Yes	14	45	No	Alive	Venous thrombosis of a lower limb

Abbreviations: VA: venoarterial; VV: venovenous; PO: postoperative day.
